# Antenatal and neonatal factors and morphology of the optic nerve head in the Northern Finland birth cohort

**DOI:** 10.1111/aos.15164

**Published:** 2022-05-10

**Authors:** Joel Pitkänen, Ilmari Leiviskä, Johanna Liinamaa, Ville Saarela

**Affiliations:** ^1^ Department of Ophthalmology, PEDEGO Research Unit and Medical Research Center University of Oulu and Oulu University Hospital Oulu Finland

**Keywords:** antenatal factors, neonatal factors, neuroretinal rim, optic disc, optic nerve head

## Abstract

**Purpose:**

The optic nerve head (ONH) is a part of the brain that can be evaluated through the transparent medium of the eye. The purpose of this study was to explore the possible correlations among the properties of the optic nerve head, maternal factors during pregnancy and neonatal parameters in a randomized sample of a birth cohort.

**Methods:**

The Northern Finland 1966 Birth Cohort has been prospectively monitored since their antenatal period. Data on pregnancy and neonatal period were collected during gestation and right after birth in 1966. A randomized sample of 3070 subjects underwent an ophthalmic assessment at the age of 46–48 years. The examination protocol included scanning laser ophthalmoscopy with the Heidelberg Retina Tomograph. The ophthalmological parameters assessed were the disc area and the neuroretinal rim volume of the ONH.

**Results:**

We found that chronic pulmonary disease of the mother (p = 0.007), the number of gestational weeks (p = 0.030) and the mother's highest measured systolic blood pressure (p = 0.035) during pregnancy had a statistically significant effect on the disc area. Smaller disc size was associated with pulmonary disease and early gestation. There was a significant difference in rim volume between genders (p < 0.001). Women had larger neuroretinal rim volumes compared to men.

**Conclusion:**

In this population‐based study, the vast majority of antenatal and neonatal factors showed no correlation with optic disc area or rim volume. Furthermore, even the factors with statistically significant correlation with ONH morphology had limited predictive value.

## Introduction

The optic nerve head (ONH) is the most distal part of the optic nerve. By 20 weeks of gestation, *circa* 50% of its growth has occurred, 75% by birth and 95% during the first year of life (Rimmer et al. 1993). During the first and beginning of the second trimester, there is an overproduction of axons in the optic nerve. Subsequently, their number decreases and stabilizes by the end of the second trimester (Provis et al. 1985). Therefore, it may be hypothesized that the size of the optic disc is influenced by the peak number of axons and the size of the cup and neuroretinal rim by the final number of axons left.

Only a few antenatal or perinatal factors affecting the size and shape of the optic nerve head have been established. Birthweight, gender and prematurity have been shown to influence the morphology of the ONH (Hellström et al. 1997; Hellström et al. 2000; Hellgren et al. 2009; Samarawickrama et al. 2009; Hackl et al. 2013; Park et al. 2013; Alshaarawi et al. 2014; Raffa et al. 2016; Åkerblom et al. 2018). Brain lesions have been associated with changes in the optic disc morphology (Jacobson et al. 1997; Hellström 1999; Jacobson et al. 2003). Foetal alcohol syndrome has been shown to change ONH morphology (Hellström 1999; Ribeiro Oliveira et al. 2007). Mother's smoking affects the retinal nerve fibre layer (RNFL) but not the ONH parameters (Pueyo et al. 2011). Racial differences in the cup‐to‐disc ratio have been shown to be present at birth (Allingham et al. 2013).

Apart from the findings above, there is limited knowledge on antenatal or neonatal factors affecting the morphology of the ONH. Furthermore, data on the effect of these factors in a randomized, population‐based sample are scarce.

The Northern Finland Birth Cohort (NFBC) offers the possibility to assess the significance of antenatal and perinatal factors affecting the ONH in a randomized population with prospective follow‐up (Nordström et al. 2021). The purpose of this study is to explore and evaluate the possible correlations among the size of the optic disc, neuroretinal rim volume and the data collected during pregnancy and birthweight, height and gender in a randomized sample of the cohort study population.

## Materials and Methods

The Northern Finland Birth Cohort 1966 (NFBC 1966) consists of subjects born in Finland's two most northern provinces in 1966. It is an unselected and geographically defined population. Qualitative and quantitative data on the cohort have been prospectively collected throughout the years (Rantakallio 1988). The original NFBC study population consisted of 12 058 subjects, covering 96% of the children born in the area.

Antenatal data were collected during visits to antenatal clinics and using a questionnaire sent to the mothers in the 1960s (Nordström et al. 2021). The antenatal parameters evaluated in the present study included mother's smoking before pregnancy and amount of smoking during the last 3 months of pregnancy, weight increase during pregnancy, number of deliveries and miscarriages and the highest systolic and diastolic blood pressure during pregnancy. Blood pressure was measured twice during pregnancy (once during months 2–4 and once during months 5–10), and the data were combined so that the higher value was chosen. The effect of general health during pregnancy was also studied. The factors included diabetes, organic heart failure, hypertension, chronic renal disease, fever, placental abruption, thyrotoxicosis, hydrops, albuminuria, urinary infection, chronic pulmonary disease and bleeding during pregnancy. The use of vitamin or iron supplements, diuretics, antibiotics, analgesics or sedatives during pregnancy was also assessed. In addition, information on the gender, placental weight, gestational weeks, birthweight and height and whether the subject is a twin or not, and the mother's age at the time of birth was collected by the maternity homes and antenatal clinics.

In 2012, when the cohort was 46 years old, 10 300 subjects living in Finland received an invitation to an extensive clinical examination of overall health. The NFBC eye study is a randomized prospective cohort study investigating the ocular health of the NFBC. Half of the cohort (*n* = 5155) were randomized to the eye screening group. Randomization was based on gender, postcode and the month of birth. Randomization was performed using Resampling Stats Software (Resampling Stats Inc., Arlington, Virginia, USA) (Saarela et al. 2013). Altogether, 3070 randomized subjects took part in the eye examination protocol. The study was conducted following the principles of the Declaration of Helsinki and was approved by the Ethical Committee of Northern Ostrobothnia Hospital District.

Ocular pathologies affecting the ONH, retina and visual fields were evaluated in a masked fashion by two ophthalmologists. There was particular interest in the evaluation of glaucomatous damage. All eyes with glaucoma suspect findings and their fellow eyes were re‐evaluated for glaucomatous damage by glaucoma specialists unaware of the results of the previous assessments. The evaluation protocol has been described previously (Saarela et al. 2013; Karvonen et al. 2019). Glaucomatous damage was not an exclusion criterion for this nonselected, randomized population.

The eye examination protocol included an assessment of the ONH. The topographic ONH parameters evaluated in the present analysis include the disc area and the rim volume. The parameters were analysed as continuous variables, and they were also divided into three groups: the smallest 5%, the largest 5% and the group in‐between. The parameters were obtained using the Heidelberg Retinal Tomograph (HRT3, Heidelberg Engineering, Heidelberg, Germany; software version 3.1.2a, Heyex 1.6.2.0). It uses confocal laser scanning imaging to measure the topography of the posterior fundus (Rohrschneider et al. 1994). The ONH parameters are calculated from the topography image by the device. The left eye of the participants was chosen for the analyses.

We tested for Pearson's correlation coefficient between the optic nerve head parameters and mother's age at the time of birth, birth length of the child, birthweight of the child, placental weight, highest systolic and diastolic blood pressure during pregnancy, increase in mother's weight during pregnancy, number of deliveries and gestational week. We used an independent *T*‐test to test the following dichotomous variables in relation to the disc area and rim volume: smoking before pregnancy, gender, birthweight over or under 2500 g, hydrops, albuminuria, urinary infection, vitamin or iron supplements, diuretics, antibiotics, analgesics or sedatives, bleeding during pregnancy and chronic pulmonary disease. Smoking during the last 3 months of pregnancy and miscarriages were variables with multiple groups. We used analysis of variance (ANOVA) to test their relationship to the disc area and rim volume. Threshold p < 0.05 was chosen for statistical significance.

ANOVA was used to test the grouped ONH parameters, mother's age and weight increase during pregnancy, the number of deliveries, birthweight and ‐length, placental weight, gestational week and the highest systolic and diastolic blood pressure. In addition, the Chi‐square test of independence was used to test the association among the grouped ONH parameters, gender, mother's chronic pulmonary disease, hydrops, albuminuria, urinary infection, vitamin or iron supplements, diuretics, antibiotics, analgesics or sedatives, bleeding during pregnancy, birthweight over or under 2500 g and smoking habits before and at the end of the pregnancy and miscarriages.

The number of diabetic mothers, mothers with organic heart failure, thyrotoxicosis, hypertension, chronic renal disease, fever, placental abruption or mothers treated for a threat of miscarriage were too low for statistical analysis. The number of subjects born as twins was also too low.

## Results

The differences between the mean disc area and rim volume were 0.0009 mm^2^ and 0.0007 mm^3^ when the study population including glaucoma cases was compared to the study population without glaucoma cases found in the NFBC Eye Study. This is due to low number of glaucoma cases (left eye cases *n* = 21). The mean rim volume of the subjects with glaucoma was 0.37 mm^3^ and the rim volume of the study population was 0.47 mm^3^. No other diseases of the ONH were found.

Smoking before or at the end of the pregnancy did not have a statistically significant correlation with the optic disc area or the rim volume of the study subjects. Mother's weight gain or age, highest diastolic blood pressure or the number of deliveries or miscarriages did not significantly correlate with the study participants' optic disc area or rim volume. (Table 1 & 2).

**Table 1:** Correlation and distribution of maternal factors, neonatal measurements and disc area.

	Pearson's correlation coefficient		
	N	R	p
Birth length	**2939**	**0.046**	**0.012**
Birthweight	2968	0.035	0.054
Gestational weeks	**2867**	**0.031**	**0.030**
Number of deliveries	894	0.018	0.592
Placental weight	2593	0.013	0.521
Mother's weight increase during pregnancy	651	−0.016	0.686
Mother's age	2939	0.004	0.809
Highest systolic blood pressure during pregnancy	**2741**	**0.040**	**0.035**
Highest diastolic blood pressure during pregnancy	2740	0.000	0.984

	*t*‐test		
	N	mean	p
Female	1645	2.168	0.085
*Male*	1336	2.198	
No Pulmonary disease	**2574**	**2.184**	**0.007**
*Pulmonary disease*	**62**	**2.020**	
No smoking before pregnancy	2364	2.184	0.501
*Smoking before pregnancy*	533	2.169	
No hydrops	1909	2.187	0.194
*Hydrops during pregnancy*	852	2.162	
No albuminuria	2525	2.171	0.772
*Albuminuria during pregnancy*	226	2.181	
No urinary infection	769	2.191	0.337
*Urinary infection during pregnancy*	60	2.130	
No vitamin or iron supplements	67	2.155	0.567
*Vitamin of iron supplements during pregnancy*	768	2.189	
No diuretics	709	2.190	0.868
*Diuretics during pregnancy*	112	2.181	
No antibiotics	740	2.195	0.392
*Antibiotics during pregnancy*	80	2.147	
No analgetics or sedatives	767	2.196	0.137
*Analgetics or sedative during pregnancy*	53	2.096	
No bleeding	793	2.195	0.363
*Bleeding during pregnancy*	30	2.114	
Birthweight > = 2500 g	2888	2.180	0.764
*Birthweight < 2500 g*	80	2.196	

	ANOVA		
	N	mean	p
*Did not smoke at end of the pregnancy*	2558	2.180	0.290
1–5 cigarettes or 1–3 pipefuls	167	2.196	
6–10 cigarettes or 4–8 pipefuls	71	2.069	
11–15 cigarettes	20	2.249	
16–20 cigarettes	18	2.309	
More than 20 cig or more than 8 pipes	5	2.276	
*0 miscarriage*	2413	2.180	0.458
1 miscarriage	352	2.199	
2 miscarriages	100	2.203	
3 or more miscarriages	39	2.078	

Factors written in bold are the ones in which statistically significant difference was found (*p* < 0.050). The italic entries are used to highlight the compared groups in *t*‐tests, for example: female vs *male*. In tables showing the results of anova‐test the italics are used to highlight the place where new comparison of groups starts, for example in Table 1: *did not smoke at the end of the pregnancy, 0 miscarriage*.

**Table 2:** Correlation and distribution of rim volume, maternal and neonatal factors

	Pearson's correlation coefficient		
	N	R	p
Birth length	2938	−0.025	0.169
Birthweight	2967	−0.006	0.733
Gestational weeks	2866	0.029	0.117
Number of deliveries	893	−0.008	0.808
Placental weight	2592	−0.013	0.511
Mother's weight increase during pregnancy	650	0.012	0.751
Mother's age	2938	−0.007	0.698
Highest systolic blood pressure during pregnancy	2738	−0.019	0.327
Highest diastolic blood pressure during pregnancy	2739	0.020	0.307

	*t*‐test		
	N	mean	p
Female	**1644**	**0.482**	**<0.001**
*Male*	**1336**	**0.445**	
No Pulmonary disease	2573	0.464	0.876
*Pulmonary disease*	62	0.461	
No smoking before pregnancy	2364	0.467	0.394
*Smoking before pregnancy*	533	0.461	
No hydrops	1908	0.466	0.588
*Hydrops during pregnancy*	852	0.462	
No albuminuria	2524	0.470	0.658
*Albuminuria during pregnancy*	226	0.465	
No urinary infection	768	0.472	0.780
*Urinary infection during pregnancy*	60	0.466	
No vitamin or iron supplements	67	0.456	0.461
*Vitamin of iron supplements during pregnancy*	767	0.472	
No diuretics	709	0.470	0.477
*Diuretics during pregnancy*	111	0.482	
No antibiotics	739	0.472	0.541
*Antibiotics during pregnancy*	80	0.460	
No analgetics or sedatives	767	0.472	0.947
*Analgetics or sedative during pregnancy*	52	0.470	
No bleeding	793	0.473	0.690
*Bleeding during pregnancy*	29	0.460	
Birthweight > = 2500 g	2887	0.466	0.360
*Birthweight < 2500 g*	80	0.449	

	ANOVA		
	N	mean	p
*Did not smoke at end of the pregnancy*	2557	0.467	0.964
1–5 cigarettes or 1–3 pipefuls	167	0.466	
6–10 cigarettes or 4–8 pipefuls	71	0.448	
11–15 cigarettes	20	0.473	
16–20 cigarettes	18	0.477	
more than 20 cig or more than 8 pipes	5	0.476	
*0 miscarriage*	2413	0.465	0.384
1 miscarriage	351	0.467	
2 miscarriages	100	0.483	
3 or more miscarriages	39	0.440	

Factors written in bold are the ones in which statistically significant difference was found (*p* < 0.050). The italic entries are used to highlight the compared groups in *t*‐tests, for example: female vs *male*. In tables showing the results of anova‐test the italics are used to highlight the place where new comparison of groups starts, for example in Table 1: *did not smoke at the end of the pregnancy, 0 miscarriage*.

There was no statistically significant correlation among albuminuria, hydrops, urinary infection or bleeding during pregnancy with the disc area or the rim volume. Furthermore, the use of vitamin or iron supplements, antibiotics, analgesics or sedatives, or diuretics during pregnancy did not significantly correlate with the disc area or the rim volume. Birthweight or placental weight did not have a statistically significant correlation with the optic disc parameters. There was no significant difference when the disc area or the rim volume of the study subjects with a birthweight under 2500 g was compared to those with a birthweight of or over 2500 g. (Table 1 & 2).

**Fig. 1:**
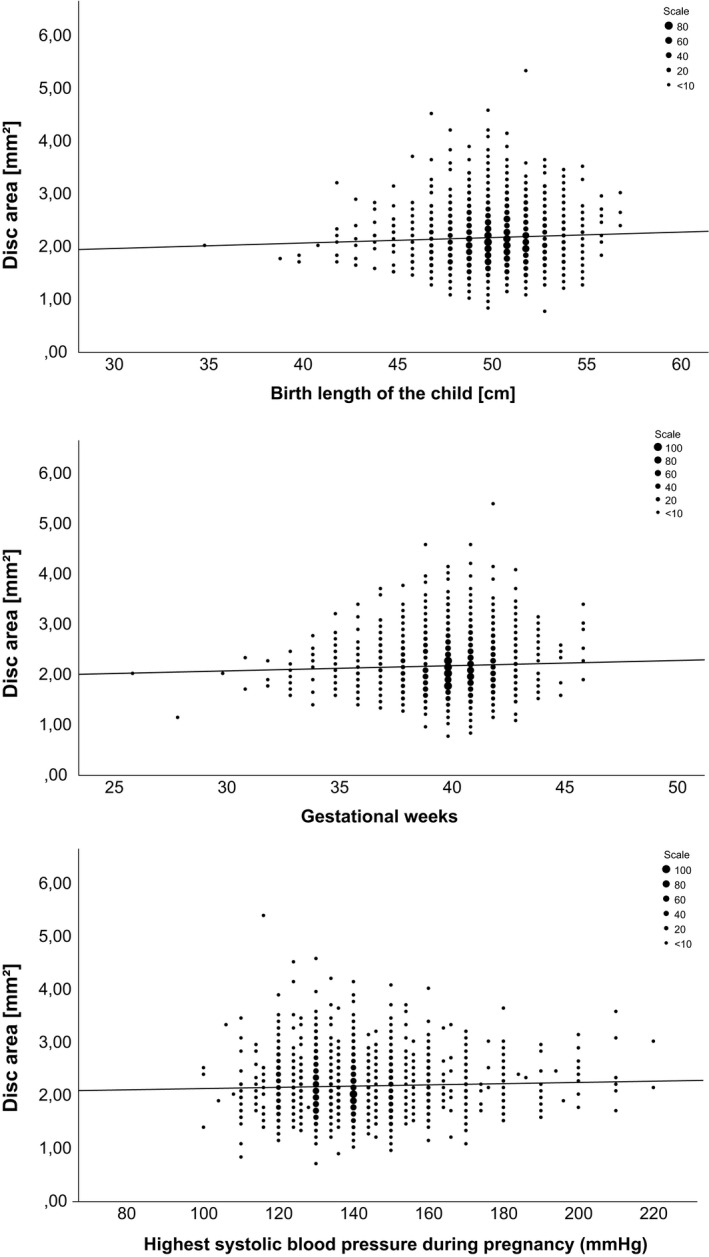
The statistically significant correlations between the ONH disc area and birth length, gestational weeks and highest systolic blood pressure during pregnancy. *Scale = number of cases are represented by each symbol.

The study subjects whose mother had a chronic pulmonary disease had smaller disc areas (mean difference = 0.164 mm^2^, p = 0.007, *t*‐test). However, there was no difference in rim volume (mean difference = 0.030 mm^3^, p = 0.876) compared to subjects whose mother did not have a chronic pulmonary disease. Gender significantly affected the rim volume (mean difference = 0.037 mm^3^, p < 0.001), but not on the disc area (mean difference = 0.030 mm^2^, p = 0.085). Women had larger neuroretinal rim volumes than men. There was a statistically significant correlation between the study subjects' birth length (R = 0.046, p = 0.012, Pearson's correlation coefficient) and the optic disc area. Nevertheless, the correlation was not statistically significant when tested separately for men (R = 0.046, p = 0.142) and women (R = 0.042, p = 0.088). Birth length and rim volume did not have a statistically significant correlation (R = ‐0.025, p = 0.169). Gestational weeks had a statistically significant correlation with the optic disc area (R = 0.031, p = 0.030) but not with the rim volume (R = 0.029, p = 0.117). The highest systolic blood pressure correlated positively with the disc area (R = 0.040, p = 0.035) but not with the rim volume (R = ‐0.019, p = 0.327). (Table 1 & 2, Fig. 1).

Women were overrepresented in the largest 5% group of rim volume and men in the smallest 5% group of rim volume. The difference was statistically significant (p < 0.001) (Table 3). The subjects whose mother had chronic pulmonary disease were overrepresented in the smallest 5% group of disc area (p = 0.020) (Table 4). None of the infant's other attributes or maternal factors had a statistically significant correlation with the grouped ONH parameters (the smallest 5%, the largest 5% and the group in‐between) (Table 5).

**Table 3:** Cross‐tabulation between gender and grouped values for rim volume.

		Female	Male	Total
Smallest 5%	Count	70 (4%)	87 (7%)	157 (5%)
	Expected Count	87 (5%)	70 (5%)	157 (5%)
5–95%	Count	1466 (89%)	1206 (90%)	2672 (90%)
	Expected Count	1474 (90%)	1197 (90%)	2672 (90%)
Largest 5%	Count	109 (7%)	43 (3%)	152 (5%)
	Expected Count	84 (5%)	68 (5%)	152 (5%)
Total	Count	1645	1336	2981
	Expected Count	1645	1336	2981

p < 0.001.

**Table 4:** Cross‐tabulation between mother's chronic pulmonary disease and grouped values for disc area.

		Chron. pulm. disease	No chron. pulm. disease	Total
Smallest 5%	Count	6 (10%)	124 (5%)	130 (5%)
	Expected Count	3 (5%)	127 (5%)	130 (5%)
5–95%	Count	54 (87%)	2317 (90%)	2371 (90%)
	Expected Count	56 (90%)	2315 (90%)	2371 (90%)
Largest 5%	Count	2 (3%)	133 (5%)	136 (5%)
	Expected Count	3 (5%)	133 (5%)	136 (5%)
Total	Count	62	2574	2636
	Expected Count	62	2574	2636

p = 0.020.

**Table 5:** Results among grouped ONH parameters, neonatal measurements and maternal factors.

	Disc area				Rim volume			
	p	μ (<5%)	μ (5–95%)	μ (>5%)	p	μ (<5%)	μ (5–95%)	μ (>5%)
Birth length (cm)	0.735	50.4	50.3	50.4	0.543	50.5	50.3	50.2
Birthweight (g)	0.899	3482	3466	3455	0.646	3501	3478	3464
Gestational weeks	0.179	40.2	40.1	40.4	0.756	40.2	40.1	40.2
Number of deliveries	0.812	2.51	2.71	2.76	0.934	2.64	2.71	2.62
Placental weight (g)	0.271	631	639	650	0.517	661	647	651
Mother's weight increase(kg)	0.099	12.1	10.2	10.5	0.294	11.5	10.3	10.6
Mother's age	0.741	27.6	27.9	28.2	0.504	27.3	28	27.9
Highest SBP during pregnancy	0.329	128.2	128.4	129	0.359	127.4	128.5	127.6
Highest DBP during pregnancy	0.719	78.8	79.1	79.4	0.546	79.3	79.1	78.8

	p	p
Female	0.066	**<0.001**
*Male*		
No Pulmonary disease	**0.020**	0.383
*Pulmonary disease*		
No smoking before pregnancy	0.481	0.682
*Smoking before pregnancy*		
No hydrops	0.743	0.818
*Hydrops during pregnancy*		
No albuminuria	0.242	0.781
*Albuminuria during pregnancy*		
No urinary infection	0.763	0.325
*Urinary infection during pregn*.		
No vitamin/iron supplements	0.234	0.795
*Vitamin/iron suppl. During pregn*.		
No diuretics	0.641	0.089
*Diuretics during pregnancy*		
No antibiotics	0.961	0.273
*Antibiotics during pregnancy*		
No analgetics or sedatives	0.147	0.940
*Analgetics/sedatives during pregn*.		
No bleeding	0.734	0.746
*Bleeding during pregnancy*		
Birthweight > = 2500 g	0.115	0.693
*Birthweight < 2500 g*		
*Did not smoke at end of the pregn*.	0.711	0.152
1–5 cigarettes or 1–3 pipefuls		
6–10 cigarettes or 4–8 pipefuls		
11–15 cigarettes		
16–20 cigarettes		
more than 20 cig or 8 pipes		
*0 miscarriage*	0.236	0.114
1 miscarriage		
2 miscarriages		
3 or more miscarriages		

Factors written in bold are the ones in which statistically significant difference was found (*p* < 0.050). The italic entries are used to highlight the compared groups in *t*‐tests, for example: female vs *male*. In tables showing the results of anova‐test the italics are used to highlight the place where new comparison of groups starts, for example in Table 1: *did not smoke at the end of the pregnancy, 0 miscarriage*.

* μ = mean, μ (<5%) = smallest 5%, μ (>5%) = largest 5%, μ (5–95%) = group in‐between, SBP/DBP = systolic/diastolic blood pressure.

## Discussion

We found that excluding mother's chronic pulmonary disease and highest measured blood pressure during pregnancy, none of the studied diseases, physiological measurements of the mother, previous miscarriages or deliveries, drugs or supplements used during pregnancy had a significant effect on the optic nerve head in a randomized birth cohort population. There was a difference in the rim volume between genders. The gestational weeks correlated positively with the disc area. Nevertheless, the measured differences and correlations were negligible at best.

We found that women had slightly larger rim volumes than men. Many studies have researched the differences in optic nerve head morphology between genders with varied results. In some studies, men's disc areas and rim areas were larger, but the difference was insignificant in other studies (Jonas et al. 1988; Ramrattan et al. 1999; Bourne et al. 2008). Abe *et al*. found that gender had no statistically significant effect on the rim volume (Abe et al. 2009).

A novel finding in this study was the smaller disc area of study subjects whose mothers had a chronic pulmonary disease during pregnancy. Intrauterine hypoxia has been shown to decrease neuron count of the developing foetus (Lawrence et al. 2019). This may affect the peak number of axon of the optic nerve. Also, a novel finding was the correlation between the highest systolic blood pressure and the ONH disc area. Study participants whose mother had higher highest measured systolic blood pressure had larger ONH disc areas. Neurodevelopment of the foetus may be affected by hypertension through a variety of biological mechanisms, e.g. *via* angiogenetic and inflammatory pathways. (Scime et al. 2021). Yet, stronger results on hypertension's effect on ONH development are needed before more detailed hypothesizing on the underlying mechanisms is warranted.

We also found that subjects born taller had larger disc areas, but when tested separately for genders, the test yielded insignificant results. This result could imply that gender affects the birth length and the optic disc area in our study population. Nevertheless, there seems to be no significant effect between birth length and the optic disc area. Samarawickrama *et al*. found that short birth length was associated with a larger cup/disc ratio in children aged 12 years (Samarawickrama et al. 2009). We did not find other studies describing associations between birth length and ONH morphology.

In our study, the birthweight did not have a statistically significant correlation with the ONH morphology (p = 0.054), but a trend towards a larger disc with increasing birthweight was present. Previous studies have found that smaller birthweight correlated with more oval optic nerve heads in premature children (Hackl et al. 2013). In very low birthweight (<1500 g) children, the optic cup area was larger, the optic rim was smaller and the optic rim/optic disc ratio smaller (Hellgren et al. 2009). In one study, the association between birthweight and optic disc morphology was insignificant in premature infants (Park et al. 2013). Low birthweight has been shown to correlate with the decreased vertical diameter of the optic nerve head (Samarawickrama et al. 2009). These results combined would imply that birthweight has only minor effect on ONH morphology.

In the present study population, the number of gestational weeks had a positive correlation with disc area. This could be because the ONH has less time for developing in subjects born prematurely. However, the correlation was feeble and explained only 0.1% (R^2^ = 0.001) of the variation of the disc area. Mixed results have been published before. Åkerblom *et al*. found that the rim area of the ONH was smaller in prematurely born children than in children born at term (Åkerblom et al. 2018). Alshaarawi *et al*. described larger rim volumes, smaller and deeper cups and increased height variation in the ONH in preterm children (Alshaarawi et al. 2014). One study showed that the child's smaller birthweight and earlier delivery correlated with more oval optic nerve heads in premature children (Hackl et al. 2013). In very low birthweight (<1500 g) children, it was found that the cup area was larger, the optic rim area smaller and optic rim/optic disc‐ratio smaller in children born prematurely when compared to subjects born full term (Hellgren et al. 2009). Hellström *et al*. found no difference in optic disc morphology between prematurely born and children born full term. They showed that lower birthweight correlated negatively with the disc area of the optic nerve head (Hellström et al. 1997). In another study, no association between optic disc parameters and birthweight was found in premature infants (Park et al. 2013). Another study focused on children born more preterm than subjects in other studies: the disc and rim areas were smaller in children born before 29 weeks of gestation when compared to ones born full term (Hellström et al. 2000). When moderate‐to‐late preterm children were compared to children born full term, no difference was found in the optic nerve head parameters (Raffa et al. 2016). It would seem that the findings are largely dependent on study design and no consistent conclusions on the effects of gestational weeks on ONH morphology can be derived.

Our study differs from previous studies on factors affecting optic disc morphology in several ways. First, our study population is relatively large and older compared to other studies in which subjects from infants to adolescents have been researched. Second, the present study has the most extensive range of maternal factors affecting the optic nerve head parameters development compared to previous studies. Third, the data collection of the antenatal and neonatal factors and ONH parameters has been performed prospectively, which improves the quality of the data. Fourth, the long period between birth and the clinical study might be considered a weakness, but the ONH size remains stable except in subjects with high myopia (Fledelius & Goldschmidt 2010).

The participation rate to the clinical examination may be considered a limitation of the study. However, a participation rate of 60%, as obtained in the present study, is considered relatively high in a population‐based cohort, especially as the same subjects have been examined repeatedly over decades. In addition, our study design is a cross‐sectional study of a prospective birth cohort, whereas previous studies use case–control study design or retrospective cohort study design (Hellström et al. 1997; Jacobson et al. 1997; Hellström 1999; Hellström et al. 2000; Jacobson et al. 2003; Ribeiro Oliveira et al. 2007; Hellgren et al. 2009; Pueyo et al. 2011; Hackl et al. 2013; Alshaarawi et al. 2014; Raffa et al. 2016; Åkerblom et al. 2018).

Despite the statistically significant correlation with ONH morphology, the correlations were weak at best for all factors tested. The low correlations may be explained by the complex, multifactorial nature of ONH development during different stages of the foetal period. ONH morphology may be influenced by numerous genetic and physiological and pathological environmental factors affecting growth and ocular and neural development. In conclusion, in our study population, the measured maternal factors and infants' attributes did not have a clinically significant effect on ONH morphology and it would seem that the development of the optic nerve head is well protected during pregnancy.
